# Hospital cleaning: past, present, and future

**DOI:** 10.1186/s13756-023-01275-3

**Published:** 2023-08-22

**Authors:** Stephanie J. Dancer

**Affiliations:** Department of Microbiology, NHS Lanarkshire & School of Applied Sciences, Edinburgh Napier University, Scotland, UK

**Keywords:** Hospital cleaning, Monitoring, Healthcare environment, Microbiological standards

## Abstract

**Introduction:**

The importance of hospital cleaning for controlling healthcare-associated infection (HAI) has taken years to acknowledge. This is mainly because the removal of dirt is inextricably entwined with gender and social status, along with lack of evidence and confusion over HAI definitions. Reducing so-called *endogenous* infection due to human carriage entails patient screening, decolonisation and/or prophylaxis, whereas adequate ventilation, plumbing and cleaning are needed to reduce *exogenous* infection. These infection types remain difficult to separate and quantitate. Patients themselves demonstrate wide-ranging vulnerability to infection, which further complicates attempted ranking of control interventions, including cleaning. There has been disproportionate attention towards endogenous infection with less interest in managing environmental reservoirs.

**Quantifying cleaning and cleanliness:**

Finding evidence for cleaning is compromised by the fact that modelling HAI rates against arbitrary measurements of cleaning/cleanliness requires universal standards and these are not yet established. Furthermore, the distinction between cleaning (soil removal) and cleanliness (soil remaining) is usually overlooked. Tangible bench marking for both cleaning methods and all surface types within different units, with modification according to patient status, would be invaluable for domestic planning, monitoring and specification.

**Aims and objectives:**

This narrative review will focus on recent history and current status of cleaning in hospitals. While its importance is now generally accepted, cleaning practices still need attention in order to determine how, when and where to clean. Renewed interest in removal and monitoring of surface bioburden would help to embed risk-based practice in hospitals across the world.

## Background

In 1974, the Committee on Infections within Hospitals of the American Hospital Association stated that, ‘the occurrence of nosocomial infection has not been related to levels of microbial contamination of air, surfaces, and fomites. So that meaningful standards for permissible levels of such contamination do not exist’ [1]. A comment in the *Journal of Hospital Infection* in 1988 broadly concurred, saying that the condition of surfaces being cleaned, frequency of cleaning and cleaning methods in UK hospitals were unlikely to have a major effect on the number of patients becoming infected [2]. It was assumed that organisms in the inanimate environment were merely “innocent bystanders,” rather than a source of patient colonization and infection [3]. Knowing the precise number of organisms present in the environment ‘…without being aware of their nature…’ would not be a relevant measure of infection risk nor would it justify the time and expense of measurement [2]. The same paper asked, ‘How clean is clean? This is predominantly a functional and aesthetic value rather than microbiological but the environment should be clean enough not to cause concern to patients and it should be microbiologically safe’ [2]. These two factors may well be connected, but they are separate entities requiring an entirely different approach.

So, the prevailing view during the late 20th century was that the environment was not thought to be important in HAI. If researchers did ponder a link between environmental surfaces and infection, then floor cleanliness was investigated rather than anything else. Inevitably, microbes found on floors (and walls) were rarely implicated as sources of infection [4, 5]. This is hardly surprising. A systematic review from 2004 examined the impact from different cleaning methods and found no difference between either cleaning procedure nor any impact on HAI rates [6]. There were just three studies in this review, mainly focusing on generic surfaces such as floors and furniture. Each tried to find a difference in HAI rates after using disinfectant or detergent for general cleaning [7–9]. A later study prospectively examined the impact of two different disinfectants on the incidence of *Clostridium difficile*–associated diarrhoea (CDAD) but neither had much impact [10]. The review established aesthetic obligations as justification for cleaning and downplayed any possible role of the surface environment as a source of infection.

This view has changed over the past twenty years. Interest in hospital cleaning has gradually increased, as careful epidemiological and molecular studies have confirmed clear links between patient infections and the healthcare environment [3, 11−13]. Patients themselves contributed towards this view since their comments on hygiene have been linked with tangible figures on hospital-acquired infection (HAIs) [14, 15]. Indeed, the UK media (rather than scientific discourse) also played a pivotal role in a renewed focus on hospital hygiene, by reporting patient complaints of visually dirty wards and infections caused by resistant hospital pathogens [16–18]. Patients and visitors put two and two together and damaging newspaper reports regarding incidents and outbreaks of methicillin-resistant *Staphylococcus aureus* (MRSA) and *C. difficile*, forced both managers and government to examine hospital cleanliness more closely. This resulted in the launch of national surveillance programmes for HAIs, after which appeared a range of different monitoring systems for hygienic practices including cleaning [19]. Such an approach has flourished on an international basis [20]. However, there remains dissent over the extent of the environmental contribution to HAI, given that patients often suffer infections emanating from their own microbial carriage [21–23]. Furthermore, the evidence base is compromised by global attitudes towards quality cleaning, which varies considerably within and between hospitals, and indeed, countries [24]. Environmental monitoring has not generally progressed further than visual inspection, and indeed, measurable standards for surface bioburden have not yet been universally accepted. In general, cleaning practises depend upon the whim of individuals, including those in managerial positions.

The aim of this review will focus on the recent history and current status of domestic cleaning in hospitals, with emphasis on general surface cleaning for clinical areas and especially sites within the patient bedspace. Decontamination of clinical/patient equipment and sterilisation of surgical instruments are not included. It is clear that cleaning practices require renewed and persistent attention in order to determine how, when and where to clean, and what is needed to embed risk-based practice into hospitals across the world [12].

### Why has it taken so long for hospital cleaning to attract attention?

**Fig. 1:**
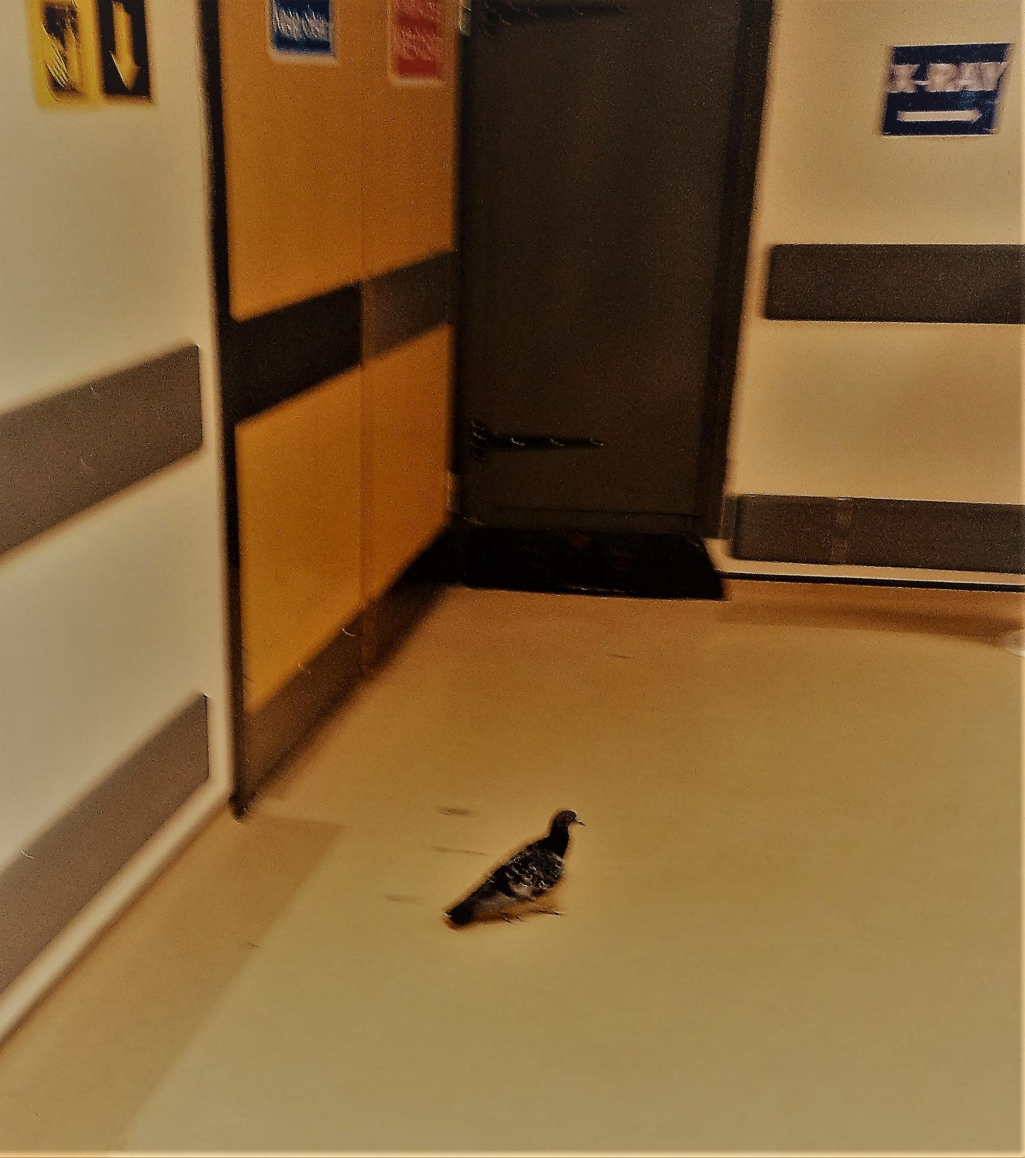
Pigeon on his way to X-ray, UK cancer hospital basement corridor, May 2023

The importance of hospital cleaning has long suffered a profound lack of recognition and there are several reasons for this. Firstly, healthcare pathogens of interest are invisible; this might be obvious to a microbiologist but not necessarily to anyone else, including healthcare workers. Pathogen presence can only be confirmed after clinical and/or environmental specimens are sent to the laboratory for processing. Cultures predominantly yield bacteria and fungi, because these are relatively straightforward to culture, but there are specific viruses and parasites associated with HAI that require specialised detection methods. Pests such as mice, rats, cockroaches, birds and Pharaoh’s ants (and associated debris) are visible but these constitute a rather different challenge for infection control (Fig. 1) [25].

Secondly, there is, of necessity, an aesthetic bias [11]. Hospitals that are visibly dirty are not acceptable to staff, patients or visitors and steps are taken to remedy this on a routine basis. Indeed, extra cleaning is always implemented during an outbreak, whether or not the outbreak organism is known to exploit an environmental niche. So instituting a study to examine the effect of quality cleaning in one unit or hospital, while disregarding practices in another would never be permitted.

Thirdly, even in the midst of an outbreak, it may be difficult to target key environmental sites simply because the ward offers such a huge surface area from which to sample. There are multiple sites available for microscopic contamination, including the air. Further compounding this, is the fact that current sampling and culturing methods do not, or cannot, always detect the pathogen of interest. This is well illustrated by the problems faced by virologists in trying to demonstrate surface contamination with viable SARS-CoV-2 [26]. Norovirus is another example. Even spores of *C. difficile* prove challenging to detect if detailed procedures are not followed in the laboratory [27].

Next, the current emphasis on evidence-based medicine insists on data from the classic randomised, controlled and double-blinded trial, in order to confirm the superiority of one product or intervention over another. For some of the reasons just stated, hospital cleaning presents difficulties in adhering to this standard demand [28]. Indeed, much of what we do in the name of infection prevention and control is also subject to similar challenges. It is not possible to randomise, or blind, implementation of many infection control activities, and certainly not during an outbreak. Outbreaks occur suddenly, with little warning, and require immediate institution of bundled interventions in order to bring transmission opportunities under control as quickly as possible. Unfortunately, we never know exactly which component of the bundle has had what effect on the natural progression of an outbreak [3].

Next, is the inadmissible fact that we do not yet have universally agreed standards for hard surface cleanliness in the healthcare environment [29]. When is a surface ‘clean’? [30]. Which level of contamination, for what surface type, in which area, ward or unit, provides assurance that there is less risk of a patient succumbing to HAI? This is also complicated by the fact that methods for sampling hard surfaces, culture and identification are varied, inaccurate, unreliable, necessarily expensive, time consuming and require microbiological expertise [31]. It is also the case that surfaces may be influenced by daily application of cleaning fluids, antimicrobial surface coatings, wear and tear, and even biofilm. A set of microbiological standards encompassing generic hand-touch sites within, and outside, near-patient areas, providing a benchmark for HAI risk would be extremely helpful, not just for infection control and domestic agencies, but as an early indication of a potential outbreak.

Next, there arises a cleaning issue for older hospitals, with poorly maintained fabric and internal fixtures [32]. It is much easier to clean intact, rather than disintegrating, surfaces on furniture, fixtures, fittings and floors. Asking staff to clean stained, damaged, torn, scratched, cracked or non-existent areas destroys incentive as well as allowing additional environmental niches for potential pathogens.

Finally, and perhaps most important of all, is the fact that cleaning is viewed through a prism of social class entwined with sexism. The removal of dirt is usually regarded as women’s work, and therefore of secondary importance [11]. Women clean because their perception of dirt and disgust clearly entices action whereas men either don’t notice a dirty environment or don’t care [33]. Unproven, and conflicted by sense of duty, religious and societal expectations, even fear, most women instinctively know that dirt removal is integral to their health as well as to those that they care for. This may be illustrated by the so-called ‘nesting’ instinct which flourishes during the later stages of pregnancy. Nature’s way, perhaps, of ensuring a clean environment for the vulnerable neonate. Sensing ‘dangerous’ dirt involves more than visual signals; it requires the detection of odour, ambience, clutter, alongside knowledge and insight, and it will foster discomfiture for the sensor without remedial action. In the community, an insidious, internalised sexism tells women that an impeccable home is a sign of her worth. This is embedded in societies across the world, intertwined with low pay; low status; and widespread social attitudes to dirt and its removal [33, 34]. Hospital cleaners are themselves ‘invisible’ on a hospital ward, just like the pathogens they seek to remove. This ‘invisibility’ is not necessarily visual, since the term can also be used as a comment on societal standing [35]. Cleaners recognise the value of what they do, but on a busy hospital ward their work is often overlooked by others, notably clinical staff.

### A brief history of hospital cleaning

When did interest in hospital cleaning first arise? While there were 18th century monographs on hospital infection describing ‘wards tainted by unwholesome effluvia’, the concept of a *clean* hospital was first pioneered by Florence Nightingale, a woman and a nurse [11, 36]. Nightingale was well aware of the spread of disease by direct or indirect contact [37]. Before this, there had been various theories encompassing so-called ‘miasma’, where diseases floated through the air over distances, and ‘contagion’, whereby person-to-person spread of disease occurred [38]. But it was Nightingale who went a step further and applied practical interventions in order to reduce ‘contagion’. While working in the Crimea, she and her nursing team reduced infection rates with basic cleaning, wound hygiene, fresh air, laundry practices and bed spacing. Her long-lasting legacy on hospital hygiene included hospital design, provision of fresh air and hygiene practices, and remained for well over a century before scientific and economic challenge [11].

Cleaning was eventually established as an HAI remedy in the late 1990’s, but there had already been interest in the role of the environment as a pathogen reservoir during the 1960’s. One article reported potential links between contaminated surfaces and community outbreaks, resolving only after introducing appropriate cleaning and disinfection regimens [39]. These outbreaks highlighted specific items such as contaminated chopping boards (salmonella) and diving masks (Gram-negative organisms and fungi), as well as surfaces in a woollen mill associated with an outbreak of cutaneous *Bacillus anthracis*. The review stated that the mill surfaces were highly important as the source of spores, and that the sampling area provided a direct correlation with the degree of hygiene and risk of disease [39]. The latter provides a prophetic statement for hospital cleaning standards first proposed in 2004 [29].

An Australian study in 1967 linked hospital-acquired *S. aureus* with persistent environmental reservoirs after phage-typing isolates from patients’ noses, wounds, air, linen and curtains [40]. Prior to this, the same authors had investigated the contribution of bedding and air to hospital-acquired staphylococcal infection [41]. The 1967 study widened the sampling scope to include curtains and found that over 20% were contaminated with patient strains. The authors could not determine whether this was due to the hands of nurses and doctors or to patients themselves. They stated that the laundry facilities did not permit the frequent changing and washing of these curtains, and of note, this is often the case nowadays.

In 1974, a London-based microbiologist called Isobel Maurer wrote a book entitled, ‘Hospital Hygiene’, dedicating an entire chapter to surface cleaning [25]. This book covered all aspects of hygiene and infection control, including management of hospital pests such as rats, mice and pigeons. Maurer wrote, ‘Some hospitals are clean; in others, cleaning standards are miserably low. There is no simple solution for the problem of a dirty hospital’. She offered a check list of solutions designed to enhance cleaning practices in hospitals, many of which remain relevant today [11]. During the 1980’s, a senior nurse at the Royal Hobart hospital in Tasmania realised the risk from burgeoning MRSA in Australia and stablished an ‘infection control cleaning’ team. Domestic services retained management of the individuals involved but the team received daily operational guidance from infection control. Healthcare-associated MRSA rates at this hospital have remained low, despite regular imports from mainland Australia [42].

Cleaning barely featured in the literature during the 1980’s but in 1993 there was a paper in an American journal describing the investigation of a cluster of mupirocin-resistant *S. aureus* on a dermatology ward [43]. The authors stated that *S. aureus* was ‘not usually associated with an environmental reservoir’, but postulated that contamination was encouraged by patients with desquamating skin conditions. This study attempted PFGE typing of strains from patients and environmental sources and showed that all mupirocin-resistant isolates had similar DNA typing patterns. The authors called for more stringent cleaning of communal areas and items implicated in the outbreak. An accompanying editorial recounted a statement from a book published in 1966: “Care should be taken to avoid the too facile assumptions that an article carrying (contaminated by) staphylococci is necessarily implicated in staphylococcal cross infections’’ [44, 45]. The general consensus was that staff hands transmitted outbreak strains from patient to patient [46]. But, no *S. aureus* was actually recovered from the hands of sampled personnel during this outbreak [43]. The editorial concluded that the role of the environment as a reservoir of the epidemic strain was unresolved. Presumably, the effect from any cleaning activity aimed at removing the reservoir would also be regarded as ‘unresolved’.

In 1996, there was a screening study centred around an MRSA patient in a side room [47]. The study showed how over 40% staff entering the room could acquire MRSA on gloved hands even if they only touched surfaces within the room rather than the patient. Unfortunately, the paper did not extrapolate the potential impact of targeted cleaning. Indeed, despite detailed epidemiological work strongly suggesting a link between the environment and patient infection, this, and other papers fail to mention the word ‘cleaning’ as the obvious solution, let alone methods, or frequencies. However, a series of letters published in 1998 described widespread environmental contamination with MRSA in UK hospitals and proposed cleaning as a clear remedy [48–50]. Earlier that year there had already been two linked articles debating the state of hospital hygiene by two nurses in the Nursing Times [51, 52]. Following these, a review on hospital cleaning emerged, after managers at a Scottish hospital tried to reduce the cleaning frequencies for ‘non-clinical’ areas directly adjoining wards [11]. The review concluded that there was little or no robust scientific evidence to support the benefits from routine cleaning other than the usual aesthetic obligations. Given that this occurred while MRSA was sweeping across the whole of the UK, the review conspired to draw together different aspects of transmission risks from the environment and any articles which supported domestic activity in hospitals [11].

The first paper to highlight links between environmental, staff and patient strains suggesting exogenous acquisition of resistant Gram-negative bacilli appeared in 1999, and this study also utilised PFGE typing to confirm potential associations [53]. The screening process recovered a range of Gram-negative genera including Pseudomonas, Enterobacter, Stenotrophomonas and Acinetobacter spp. from hand-touch sites, some of which were indistinguishable from patient isolates. Two years later, there was an MRSA outbreak on a male urological ward in England which introduced and reviewed the impact of additional cleaning [54]. The results suggested that despite all the usual interventions implemented in response to the outbreak, it wasn’t until cleaning hours were doubled that the outbreak receded. The authors considered the costs incurred and found that balancing cost of infections against the cost of extra cleaning provided an estimated cost benefit of £27,786 for the 6-month outbreak in 2000. This was the first paper to examine domestic expenditure against the cost of hospital-acquired infection. Following this, came a report of another outbreak in an English hospital, this time caused by *Acinetobacter baumanniii* in a neurosurgical intensive care unit [55]. During efforts to control the outbreak, the authors noticed a significant correlation between the number of *A. baumannii* isolates from monthly surface screening and the number of patients with *A. baumannii* colonization or infection in the same calendar month (P < 0.004). Enhanced cleaning with 1000 ppm sodium hypochlorite reduced the number of environmental isolates and cases. The authors made it quite clear that high standards of cleaning were integral to controlling this outbreak [55].

While there was increasing recognition of pathogen reservoirs in the healthcare environment, with cleaning as the obvious solution, not all infection control professionals were convinced. The evidence base, along with many other infection prevention activities, was circumstantial at best, and non-existent at worst. It seemed that the only way to raise awareness of the cleaning effect was to establish microbiological standards for surface bioburden in order to be able to measure impact. Utilising tangible counts would enable researchers to model bioburden against a range of parameters, including cleaning and decontamination methods, in order to find the evidence that was lacking [29].

### Measuring microbial bioburden on surfaces

During the early 2000’s, several professional bodies in the UK published standards or audits regarding environmental cleanliness in hospitals [56, 57]. Without numerical measures, however, evaluating the quality of hospital cleaning and cleanliness was limited. These, and other national guidelines, could only propose a range of visual indices, which do not necessarily correspond with microbiological risk [58]. Since cleaning could be a cost-effective method of controlling HAI, it needed investigation as a scientific process with measurable outcome. To achieve this, it was thought necessary to adopt an integrated and risk-based approach, which would include preliminary visual assessment, rapid sensitive tests for organic soil and microbiological sampling [58]. Such an approach had already been established by the food industry to manage cleaning practices in a cost-effective manner [59]. There was also an index of microbial air contamination (IMA) established for healthcare environments at risk, with maximum acceptable levels for different classes of contamination [60]. Even recreational waters are subject to analysis for microbial indicators of human sewage and corresponding health risk [61]. Clearly, if bioburden on hospital surfaces could be quantitated; monitored; and modelled against cleaning activities, staffing, occupancy and/or infection rates, among other variables, then the removal of dirt constituted a science in its own right [62].

**Fig. 2:**
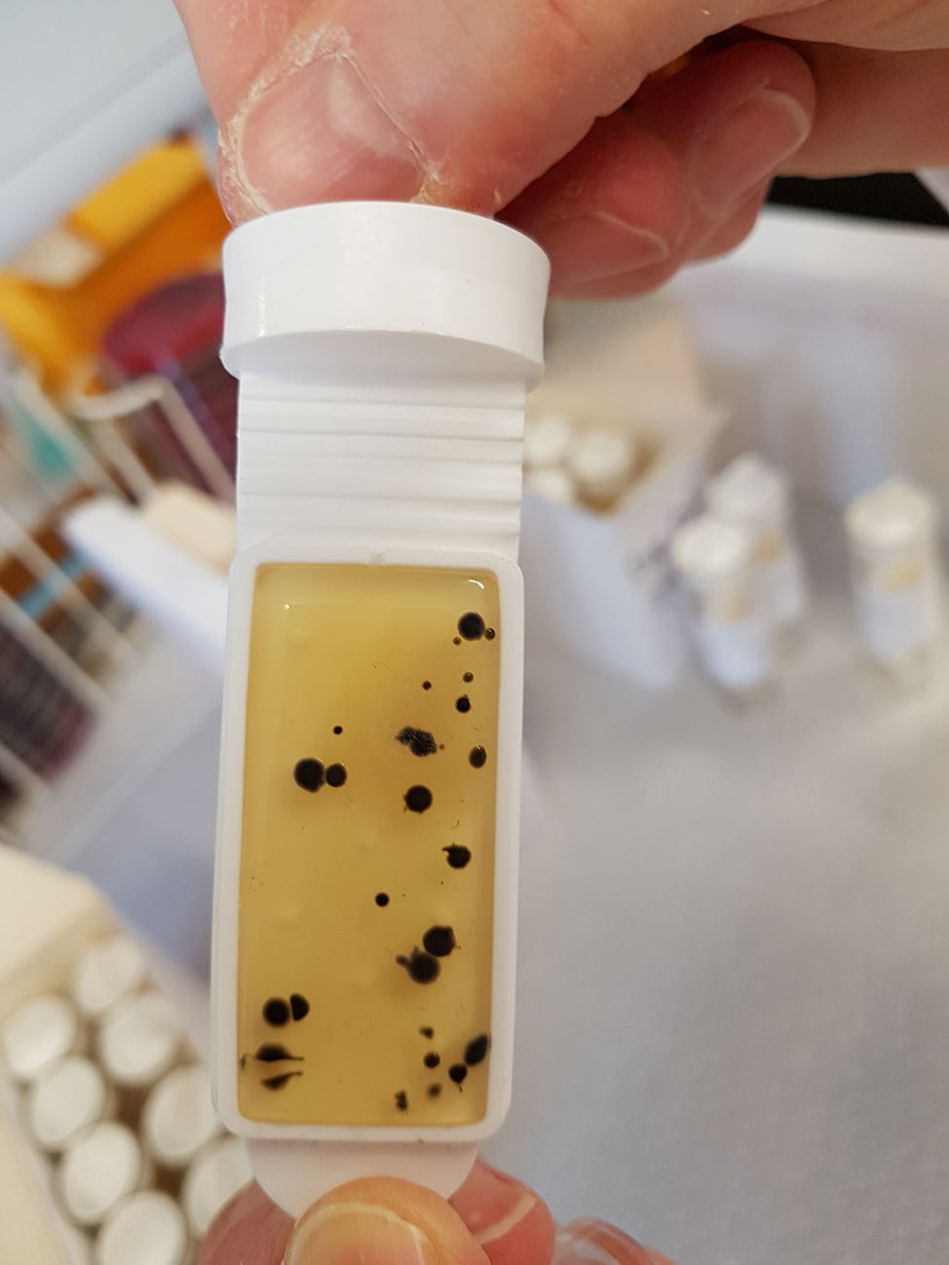
Agar covered dipslide showing < 5 cfu/cm^2^

Original microbiological standards comprised two proposals: first, the identification of an indicator organism of potential high-risk to patients (< 1 cfu/cm^2^) from hard surfaces, and secondly, the quantitative assessment of organisms recovered from a hand contact site, regardless of identity (aerobic colony count (ACC) < 5 cfu/cm^2^) (Fig. 2) [29].

Finding of ≥ 5 cfu/cm^2^ from a hand contact surface, whatever the identity of isolates, indicates that there might be an increased risk of infection for the patient in that environment. This should generate an evaluation of cleaning/disinfection practices and frequencies based on three suppositions: first, an increased microbial burden suggests that there has been insufficient cleaning. This would increase the chances of finding a pathogen. Second, a heavy microbial burden may mask the finding of a pathogen. Third, a heavy concentration of specific organisms implies an increased chance of finding an epidemiologically related pathogen, e.g. coagulase-negative staphylococci and *S. aureus*.

Given that it takes as little as 5 cfu of *S. aureus* to initiate infection, the choice of 1cm [2] as the surface area standard was deliberate [29, 63, 64]. The area of the top of an adult digit is close to 1cm [2], with that of the thumb even larger. Sampling visually clean hands repeatedly furnish multiple cfu of skin organisms but few pathogens including *S. aureus* [65].

Are hospital cleanliness standards useful? The answer to this is a generic ‘yes’, given that a range of microbiological benchmarks have been used to assess disinfectants; cleaning practices; cleaning interventions; automated devices; and antimicrobial surfaces, and surface counts can be modelled against hand-touch frequency; HAI rates; air counts; cleaning efficiencies; cleaning frequencies and additional methods for monitoring surface cleanliness. [13, 66−84] However, the choice of microbiological standards depends on multiple factors, so that every healthcare institution should decide on the standard that works for them. Once implemented, the data can be collected over time and analysed against according to HAI risk. Every hospital is different and only by analysing long term trends will relevant indicators or triggers for likely infection incidents and even outbreaks become apparent [31]. Universal hard surface standards require robust background data as well as consensus over cleaning practises.

### Current status of hospital cleaning and cleaners

Since work showing that patients in newly cleaned side-rooms have an increased risk of acquiring the same pathogen as a previous occupant, there has been increased support for the environmental role in HAI. In fact, this arguably constitutes the best justification that we have for hospital cleaning [85]. One rarely knows exactly how pathogens reach patients, however, even if we have mounting evidence for favoured environmental reservoirs. As infection control staff know only too well, ‘tracing an infection to a specific exposure is challenging’ [86]. There are a dozen or more ways a microbe travels between people and surfaces (and back again), and reservoirs include all hand-touch sites, as well as sites which cannot be, or are never touched (air vents, airborne microbes following sudden draughts, etc.). Hospital air itself constitutes a potential reservoir, although clearly under investigated at present [71]. Added to this is continued uncertainty from genotyping, which is supposed to ratify links between patients and the environment. It may not, because without detailed epidemiology, we cannot discern the direction of travel [87]. Furthermore, clonal spread of a particular pathogen can be so prolific that sequencing may not necessarily distinguish the nature of spread in infection incidents or the ever present possibility of a long-term outbreak grumbling along beneath the alert threshold [22, 88]. More research utilising careful and detailed epidemiology, supported by genotypic data, will help to cement the evidence base for a hitherto poorly studied science [28, 89].

Cleaning methods deserve hauling into the 21st century, since buckets and mops, cloths, dusters, dustpan, brushes and broom, remain the staple armoury for cleaners since the time of Florence Nightingale. We do have impregnated wipes available, at a cost; and vacuum cleaners, scrubbing machines and automated decontamination equipment, also at a cost. But the most effective cleaning practices, tailored to patient risk, are still a mystery. How exactly should surfaces be cleaned? Where should cleaning be prioritised? How often? What approach is needed for clinical areas accommodating highly vulnerable patients? Even the debate over detergents vs. disinfectants has not yet been resolved [6]. A sterile environment is not achievable, or if it is (after exposure to powerful microbicides), then it does not remain sterile for long. There exists a so-called environmental microbiome, which is disrupted every time a surface is cleaned [90, 91]. Just as for the human gut, when patients suffer overgrowth of *Clostridium difficile* following antibiotic therapy, exposure to powerful disinfectants removes resident flora on environmental surfaces, leaving vast acres of space available for other microbes to contaminate. These disinfectants remove susceptible microbes, so that only those able to withstand microbiocidal effect will remain. The amount and/or type of recontamination may be worse than what was there in the first place [69]. Wiping alone, or with water or detergent, is much less likely to damage the surface ecosystem. Hence, support for the environmentally friendly view, that we should seek to routinely remove pathogens from surfaces rather than try to obliterate them [92].

Even if healthcare cleaning has achieved global acknowledgement, the very people who perform it remain unrecognized and undervalued for the most part. Many of them receive little or no training for what they do, and they lack the career framework, structure and progression enjoyed by most other professions [93]. There are fewer opportunities for advancement in housekeeping positions, often compounded by language and literacy problems. The current status of cleaning personnel, depicted by lower pay scales and basic conditions, does not necessarily reflect the physical cleaning effort and personal risks required to protect patients from hospital pathogens. People who clean are regularly confronted by risk of injury, poisoning, or scalding from equipment and fluids, as well as infection risks from exposure to facilities and occupants with transmissible pathogens. The dichotomy between increasing attention towards a clean environment and the social and professional level of those who make it happen has widened considerably. Therein is a balance that should be redressed as we contemplate the ever increasing risk of antimicrobial resistance and untreatable infection. Cleaners constitute the front line in the war against multidrug-resistant microbial pathogens.

### Future outlook for hospital cleaning

There remain issues regarding hospital cleaning despite its undoubted importance for both aesthetic appearances and infection control. One of the most pertinent is to establish some form of training and advancement framework for people who clean hospitals, and indeed, other public venues [94]. Removing dirt is not quite as simple as it sounds. There are numerous methods for wiping, mopping, dusting and decontaminating surfaces to start with, and ever increasing product choice, including automated devices and novel disinfectants [74, 92, 95, 96]. Indeed, a risk-based approach to cleaning in the healthcare environment is still in its infancy. The is perhaps because a ward, rather than a household or office, is an unnatural and unpredictable environment, so that obvious reservoirs and transmission vectors themselves can be subjugated by microbicides, obscure contamination and unlikely vectors. For these reasons, an all eventuality approach is currently employed alongside expected aesthetic obligations. It will take a paradigm shift to set up practices based on clinical risk and the educational base required for implementation. Perhaps the first step should be to extend guidelines and policies from basic surface cleaning in hospitals to specialised cleaning tasks, such as clinical and electrical equipment, cleanrooms and high-level isolation units. Experience and qualification in such duties would help enhance the status of domestic personnel, which should be reflected by salary and career progression [94].

Standard operating procedures (SOPs) for cleaning and decontamination are evident in most hospitals. They are supposedly tailored to clinical and non-clinical areas, but there are major disparities even within hospitals, as well as between hospitals, and certainly between hospitals in different countries [24, 93]. While better resourced hospitals debate the frequency of bed space cleaning, for example, less well-off hospitals struggle to access clean water, let alone sufficient domestic staff [70, 93, 97].

**Fig. 3:**
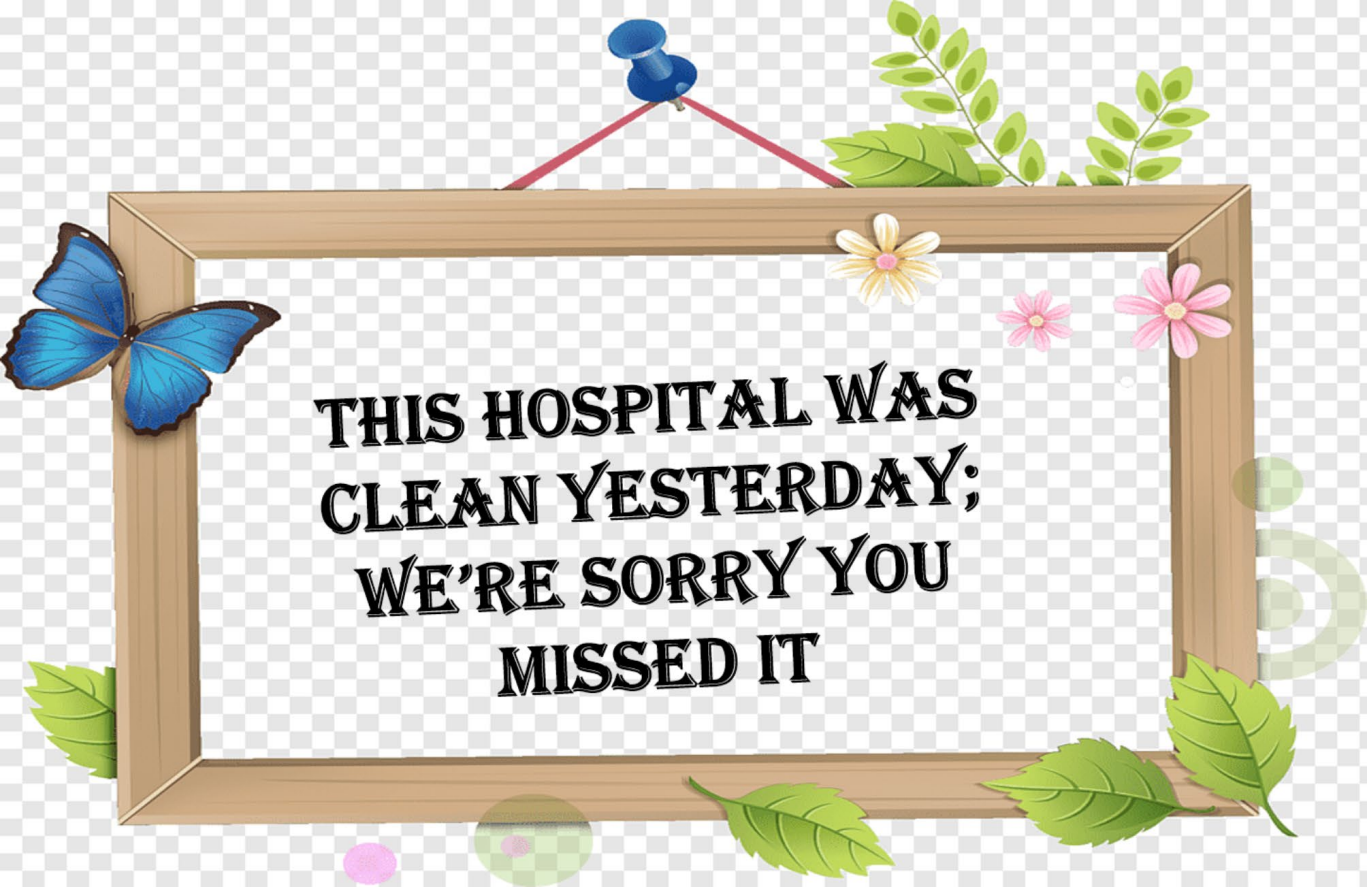
Comment on tenuous status of hospital cleanliness

Establishing some universal standards for surface cleanliness would undoubtedly help focus attention on what cleaning actually is, and what it does. As for SOPs, these should reflect type of hospital, unit, patient vulnerability and infection risk. Working towards an evidence-based benchmark should encompass routine cleaning practices, cleaning products, sampling methods and laboratory process [31, 83]. As already mentioned, it is entirely appropriate for each institution to decide on the level of cleanliness deemed appropriate for its patients therein, with long term monitoring to establish range and trends of bioburden (Fig. 3).

Sampling and processing methods need to be taken into account, since dipslide sampling may yield different quantitative data than RODAC plates or sponge sampling. Collection of environmental surveillance data over time would allow modelling against HAI rates and even predict cross-infection incidents and outbreaks. Given that some countries espouse detergent-based cleaning as opposed to a cocktail of disinfectants, then these standards will undoubtedly require flexibility and validation. Cleaning practices (especially those using microbiocidal agents) undoubtedly influence regular surface bioburden [90].

The burgeoning science of hard surface biofilm represents a potential threat to SOPs and standards, given that superficial wiping might only remove free-floating planktonic organisms, and not the entire microbiological village hidden within surface crevices [98]. Failure to disrupt biofilm does not necessarily have to invalidate this type of manual cleaning, however, since it should be possible to determine relative risk from appropriate cleaning frequencies [99]. Timely physical removal of newly liberated pathogens can be achieved while leaving the biofilm structure relatively intact. At present, optimal cleaning frequencies for all surface types and different areas of clinical risk in the healthcare environment remain unknown. Perhaps seeking complete obliteration of hard surface biofilm in our hospitals might be viewed as disproportionate, let alone time-wasting and expensive. Indeed, excessive use of disinfectants, enzymes and physical force could create additional risk, since Nature abhors a vacuum and will fill it up if she can [90].

It is likely that business and cleaning industries will continue to produce innovative products and equipment aimed at removing or neutralising dirt from the environment. Industry and infection control staff appear to be fixated on automated decontamination devices at present but the associated problems and huge cost of these, will ultimately deter managers and poorly resourced hospitals looking for simpler solutions [96]. Whatever novel device, antimicrobial surface or magic cleaning product might suddenly appear, hospitals can never discard the cleaning workforce [91, 100]. Emptying bins, replenishing linen and towels, cleaning toilets and rubbish retrieval from floors and other surfaces circumvent widespread installation of automated devices, at least for the moment [91].

Finally, in the wake of the COVID-19 pandemic, there is a new infection paradigm to consider and that concerns pathogen dissemination through the air. Most airborne particles eventually fall to the ground or any intervening surface except for the tiniest particles. This means that particle content in indoor air is a subset of what may be found on surfaces [71]. So there is a pressing need to consider methods for ‘cleaning’ indoor air – and maybe even the provision of risk-based standards for clean air in all public venues, including hospitals [101]. Cleaning the air in hospitals, whether through filtration, disinfection or fresh air replacement, will have a beneficial effect on deposited surface bioburden, including pathogens [102].

## Conclusion

Has hospital cleaning finally ‘come of age’, rather than trailing behind more popular infection control activities, such as hand hygiene? [89]. One would like to think so. The balance between hand hygiene and cleaning hand-touch sites should be equal and opposite but domestic duties have not yet generated the global flag waving witnessed for hand hygiene [103]. Hands have to touch something to function, and if the surfaces they touch are contaminated, then attempts at keeping hands clean are automatically invalidated. Furthermore, it is a lot easier to ratify good cleaning than it is to sustain hand hygiene compliance. Hospital cleaning does not have to depend upon personal whim. It does require evidence-based surface standards, however, along with training, regular monitoring and education for all domestic staff. Recognising the value of a clean hospital should benefit cleaners themselves, with elevated status and corresponding salary scales [64, 94, 104].

There is no doubt that antimicrobial chemotherapy and vaccines were the 20th century antidote to infection. However, the 21st century needs to broaden its scientific attitude to infection, and that is achieved by a deeper understanding of pathogen transmission. If we cannot treat infection, we should at least try to prevent pathogens from reaching patients in the first place. Knowing where the pathogens are, and how they spread, allows clinicians, academics and commercial entities to devise practices and technology aimed at protecting people both inside, and outside, the healthcare environment. Removing dirt from surfaces, whether visible or not, is fundamental to good health [105]. It is time for cleaning, still the Cinderella of infection control, to step into the spotlight [11].

## Data Availability

Literary references taken from peer-reviewed journals and books; all figures are author’s own.

## Declarations

### Authors’ information (optional)

Stephanie J. Dancer is a Consultant Microbiologist in NHS Lanarkshire, and Professor of Microbiology at Edinburgh Napier University, Scotland, UK.

## References

[1] Maki DG, Alvarado CJ, Hassemer CA, Zilz MA (1982). Relation of the inanimate hospital environment to endemic nosocomial infection. N Engl J Med.

[2] Collins BJ. The hospital environment: how clean should a hospital be? J Hosp Infect 1988; 11 Suppl A:53 – 6. 10.1016/0195-6701(88)90166-1 2896746

[3] Hota B (2004). Contamination, disinfection, and cross-colonization: are hospital surfaces reservoirs for nosocomial infection?. Clin Infect Dis.

[4] Ayliffe GA, Collins BJ, Lowbury EJ, Babb JR, Lilly HA (1967). Ward floors and other surfaces as reservoirs of hospital infection. J Hyg (Lond).

[5] Ayliffe GA, Collins BJ, Lowbury EJ (1966). Cleaning and disinfection of hospital floors. Br Med J.

[6] Dettenkofer M, Wenzler S, Amthor S, Antes G, Motschall E, Daschner FD (2004). Does disinfection of environmental surfaces influence nosocomial infection rates? A systematic review. Am J Infect Control.

[7] Dharan S, Mouruga P, Copin P, Bessmer G, Tschanz B, Pittet D (1999). Routine disinfection of patients’ environmental surfaces: myth or reality?. J Hosp Infect.

[8] Danforth D, Nicolle LE, Hume K, Alfieri N, Sims H (1987). Nosocomial infections on nursing units with floors cleaned with a disinfectant compared with detergent. J Hosp Infect.

[9] Daschner F, Rabbenstein G, Langmaack H (1980). Flächendekontamination zur Verhütung und Bekämpfung von Krankenhausinfektionen. Bewertung verschiedener Massnahmen [Surface decontamination in the control of hospital infections: comparison of different methods]. Dtsch Med Wochenschr.

[10] Mayfield JL, Leet T, Miller J, Mundy LM (2000). Environmental control to reduce transmission of *Clostridium difficile*. Clin Infect Dis.

[11] Dancer SJ (1999). Mopping up hospital infection. J Hosp Infect.

[12] Dancer SJ (2009). The role of environmental cleaning in the control of hospital-acquired infection. J Hosp Infect.

[13] Dancer SJ, White LF, Lamb J, Girvan EK, Robertson C (2009). Measuring the effect of enhanced cleaning in a UK hospital: a prospective cross-over study. BMC Med.

[14] Kaldenberg D, Trucano M. The relationship between patient perceptions of hospital practices and facility infection rates: Evidence from Pennsylvania hospitals. Patient Safety & Quality Healthcare online 2007; Aug 22. Comment at: https://www.psqh.com/analysis/safety-and-satisfaction-where-are-the-connections/.

[15] Greaves F, Pape UJ, King D, Darzi A, Majeed A, Wachter RM, Millett C (2012). Associations between web-based patient ratings and objective measures of hospital quality. Arch Intern Med.

[16] Washer P, Joffe H (2006). The “hospital superbug”: social representations of MRSA. Soc Sci Med.

[17] Washer P, Joffe H, Solberg C (2008). Audience readings of media messages about MRSA. J Hosp Infect.

[18] Brazier JS (2008). *Clostridium difficile*: from obscurity to superbug. Br J Biomed Sci.

[19] Reilly J, Mullings A (2017). The national agenda for Healthcare Associated infection, antimicrobial resistance and infection Prevention and Control in Scotland: structures, current priorities and programmes. J Infect Prev.

[20] Storr J, Twyman A, Zingg W, Damani N, Kilpatrick C, Reilly J, Price L, Egger M, Grayson ML, Kelley E, Allegranzi B, WHO Guidelines Development Group (2017). Core components for effective infection prevention and control programmes: new WHO evidence-based recommendations. Antimicrob Resist Infect Control.

[21] Harbarth S, Maiwald M, Dancer SJ (2013). The environment and healthcare-acquired infections: why accurate reporting and evaluation of biological plausibility are important. Infect Control Hosp Epidemiol.

[22] Dancer SJ, Adams CE, Smith J, Pichon B, Kearns A, Morrison D (2019). Tracking *Staphylococcus aureus* in the intensive care unit using whole-genome sequencing. J Hosp Infect.

[23] Myers F. Letter to the, editor. *Am J Infect Control* 2023 Feb 1: S0196-6553(23)00045 – 7.

[24] Kenters N, Gottlieb T, Hopman J, Mehtar S, Schweizer ML, Tartari E, Huijskens C, Voss EGW, ISAC working group Infection Prevention and (2018). An international survey of cleaning and disinfection practices in the healthcare environment. J Hosp Infect.

[25] Maurer IM (1974). Hospital Hygiene.

[26] Otter JA, Zhou J, Price JR, Reeves L, Zhu N, Randell P, Sriskandan S, Barclay WS, Holmes AH (2023). SARS-CoV-2 surface and air contamination in an acute healthcare setting during the first and second pandemic waves. J Hosp Infect.

[27] Ali S, Muzslay M, Wilson P (2015). A novel quantitative sampling technique for detection and monitoring of *Clostridium difficile* contamination in the clinical environment. J Clin Microbiol.

[28] Dancer SJ, Inkster T (2022). One size does not fit all: why infection prevention is difficult to randomize or control. J Hosp Infect.

[29] Dancer SJ (2004). How do we assess hospital cleaning? A proposal for microbiological standards for surface hygiene in hospitals. J Hosp Infect.

[30] Al-Hamad A, Maxwell S (2008). How clean is clean? Proposed methods for hospital cleaning assessment. J Hosp Infect.

[31] Dancer SJ (2015). Pitfalls in microbiological sampling of the healthcare environment. A response to “evaluating a new paradigm for comparing surface disinfection in clinical practice. Infect Control Hosp Epidemiol.

[32] Maurer IM. Hospital Hygiene, 3rd edition. Wright PSG, Bristol, 1985.

[33] Curtis V, Biran A (2001). Dirt, disgust, and disease. Is hygiene in our genes?. Perspect Biol Med.

[34] Giménez-Nadal JI, Mangiavacchi L, Piccoli L (2019). Keeping inequality at home: the genesis of gender roles in housework. Labour Econ.

[35] Yanke E, Moriarty H, Carayon P, Safdar N (2021). ’the Invisible Staff”: a qualitative analysis of Environmental Service Workers’ perceptions of the VA *Clostridium difficile* Prevention Bundle using a human factors Engineering Approach. J Patient Saf.

[36] Selwyn S (1991). Hospital infection: the first 2500 years. J Hosp Infect.

[37] Nightingale F. Notes on nursing: what it is and what it is not, D. Appleton & Co, London, UK, 1860.

[38] Jimenez JL, Marr LC, Randall K, Ewing ET, Tufekci Z, Greenhalgh T (2022). What were the historical reasons for the resistance to recognizing airborne transmission during the COVID-19 pandemic?. Indoor Air.

[39] Sanborn WR (1963). The relation of surface contamination to the transmission of disease. Am J Public Health Nations Health.

[40] Rountree PM, Beard MA, Loewenthal J, May J, Renwick SB (1967). Staphylococcal sepsis in a new surgical ward. Br Med J.

[41] Rountree PM, Beard MA (1962). Observations on the distribution of Staphylococcus aureus in the atmosphere of a surgical ward. J Hyg (Lond).

[42] Coombs GW, Daley DA, Thin Lee Y, Pearson JC, Robinson JO, Nimmo GR, Collignon P, Howden BP, Bell JM, Turnidge JD. ; Australian Group on Antimicrobial Resistance. Australian *Staphylococcus aureus* Sepsis Outcome Programme annual report, 2014. *Commun Dis Intell Q Rep* 2016; 40(2): E244-54. 10.33321/cdi.2016.40.20 27522136

[43] Layton MC, Perez M, Heald P, Patterson JE (1993). An outbreak of mupirocin-resistant *Staphylococcus aureus* on a dermatology ward associated with an environmental reservoir. Infect Control Hosp Epidemiol.

[44] Barg NL (1993). Environmental contamination with *Staphylococcus aureus* and outbreaks: the cause or the effect?. Infect Control Hosp Epidemiol.

[45] Williams R, Blowers R, Garrod L, Shooter R (1966). Staphylococcal infections: introduction. Hospital infection causes and Prevention.

[46] Rammelkamp C, Morttimer E, Wolinsky E (1964). Transmission of streptococcal and staphylococcal infections. Amz Intern Med.

[47] Boyce JM, Potter-Bynoe G, Chenevert C, King T (1997). Environmental contamination due to methicillin-resistant *Staphylococcus aureus*: possible infection control implications. Infect Control Hosp Epidemiol.

[48] Blythe D, Keenlyside D, Dawson SJ, Galloway A (1998). Environmental contamination due to methicillin resistant *Staphylococcus aureus*. J Hosp Infect.

[49] Marshall B, Sen RA, Chadwick PR, Keaney MGL (1998). Environmental contamination of a new general Surgical Ward. J Hosp Infect.

[50] Stacey A, Burden P, Croton C, Jones E (1998). Contamination of television sets by methicillin-resistant *Staphylococcus aureus* (MRSA). J Hosp Infect.

[51] Hempshall P, Thomson M (1998). Dirt Alert. Nurs Times.

[52] Hempshall P, Thomson M (1998). Grime Watch. Nurs Times.

[53] D’Agata EM, Venkataraman L, DeGirolami P, Samore M (1999). Molecular epidemiology of ceftazidime-resistant gram-negative bacilli on inanimate surfaces and their role in cross-transmission during nonoutbreak periods. J Clin Microbiol.

[54] Rampling A, Wiseman S, Davis L, Hyett AP, Walbridge AN, Payne GC, Cornaby AJ (2001). Evidence that hospital hygiene is important in the control of methicillin-resistant *Staphylococcus aureus*. J Hosp Infect.

[55] Denton M, Wilcox MH, Parnell P, Green D, Keer V, Hawkey PM, Evans I, Murphy P (2004). Role of environmental cleaning in controlling an outbreak of *Acinetobacter baumannii* on a neurosurgical intensive care unit. J Hosp Infect.

[56] A clean bill. of health? Auditor General, Audit Scotland, April; 2000.

[57] National standards of cleanliness for the NHS., NHS Estates, April; 2001.

[58] Griffith CJ, Cooper RA, Gilmore J, Davies C, Lewis M (2000). An evaluation of hospital cleaning regimes and standards. J Hosp Infect.

[59] Moore G, Griffith CJ (2002). A comparison of surface sampling methods for detecting coliforms on food contact surfaces. Food Microbiol.

[60] Pasquarella C, Pitzurra O, Savino A (2000). The index of microbial air contamination. J Hosp Infect.

[61] World Health Organization Assessment of risk. and risk management for water-related infectious disease. in: Fewtrell L. Bartram J. 1st edn. World Health Organization, London 2001.

[62] Dancer SJ (2012). Hospital cleanliness: establishing a new science. J Hosp Infect.

[63] Noble WC (1981). Microbiology of human skin.

[64] Dancer SJ (2008). Importance of the environment in meticillin-resistant *Staphylococcus aureus* acquisition: the case for hospital cleaning. Lancet Infect Dis.

[65] Trick WE, Vernon MO, Hayes RA, Nathan C, Rice TW, Peterson BJ, Segreti J, Welbel SF, Solomon SL, Weinstein RA (2003). Impact of ring wearing on hand contamination and comparison of hand hygiene agents in a hospital. Clin Infect Dis.

[66] White LF, Dancer SJ, Robertson C, McDonald J (2008). Are hygiene standards useful in assessing infection risk?. Am J Infect Control.

[67] Adams CE, Smith J, Watson V, Robertson C, Dancer SJ (2017). Examining the association between surface bioburden and frequently touched sites in intensive care. J Hosp Infect.

[68] Bogusz A, Stewart M, Hunter J (2013). How quickly do hospital surfaces become contaminated after detergent cleaning?. Healthc Infect.

[69] Stewart M, Bogusz A, Hunter J, Devanny I, Yip B, Reid D, Robertson C, Dancer SJ (2014). Evaluating use of neutral electrolyzed water for cleaning near-patient surfaces. Infect Control Hosp Epidemiol.

[70] Gon G, Kabanywanyi AM, Blinkhoff P, Cousens S, Dancer SJ, Graham WJ, Hokororo J, Manzi F, Marchant T, Mkoka D, Morrison E, Mswata S, Oza S, Penn-Kekana L, Sedekia Y, Virgo S, Woodd S, Aiken AM (2021). The clean pilot study: evaluation of an environmental hygiene intervention bundle in three Tanzanian hospitals. Antimicrob Resist Infect Control.

[71] Smith J, Adams CE, King MF, Noakes CJ, Robertson C, Dancer SJ (2018). Is there an association between airborne and surface microbes in the critical care environment?. J Hosp Infect.

[72] Casini B, Tuvo B, Scarpaci M, Totaro M, Badalucco F, Briani S, Luchini G, Costa AL, Baggiani A (2023). Implementation of an environmental cleaning protocol in Hospital critical areas using a UV-C disinfection Robot. Int J Environ Res Public Health.

[73] Schmidt MG, Attaway HH, Sharpe PA, John J, Sepkowitz KA, Morgan A, Fairey SE, Singh S, Steed LL, Cantey JR, Freeman KD, Michels HT, Salgado CD (2012). Sustained reduction of microbial burden on common hospital surfaces through introduction of copper. J Clin Microbiol.

[74] Gon G, Dansero L, Aiken AM, Bottomley C, Dancer SJ, Graham WJ, Ike OC, Lewis M, Meakin N, Okafor O, Uwaezuoke NS, Okwor TJ (2022). A better disinfectant for low-resourced hospitals? A Multi-Period Cluster Randomised Trial comparing Hypochlorous Acid with Sodium Hypochlorite in nigerian hospitals: the EWASH Trial. Microorganisms.

[75] Cadogan K, Bashar S, Magnusson S, Patidar R, Embil J, Gawaziuk JP, Gawthrop M, Liu S, Kumar A, Logsetty S (2021). Assessment of cleaning methods on bacterial burden of hospital privacy curtains: a pilot randomized controlled trial. Sci Rep.

[76] Inkinen J, Ahonen M, Iakovleva E, Karppinen P, Mielonen E, Mäkinen R, Mannonen K, Koivisto J (2020). Contamination detection by optical measurements in a real-life environment: a hospital case study. J Biophotonics.

[77] Vikke HS, Giebner M, Kolmos HJ (2018). Prehospital infection control and prevention in Denmark: a cross-sectional study on guideline adherence and microbial contamination of surfaces. Scand J Trauma Resusc Emerg Med.

[78] Mulvey D, Redding P, Robertson C, Woodall C, Kingsmore P, Bedwell D, Dancer SJ (2011). Finding a benchmark for monitoring hospital cleanliness. J Hosp Infect.

[79] Cooper RA, Griffith CJ, Malik RE, Obee P, Looker N (2007). Monitoring the effectiveness of cleaning in four british hospitals. Am J Infect Control.

[80] Ferreira AM, de Andrade D, Rigotti MA, de Almeida MT, Guerra OG, dos Santos Junior AG (2015). Assessment of disinfection of hospital surfaces using different monitoring methods. Rev Lat Am Enfermagem.

[81] Ibfelt T, Foged C, Andersen LP (2014). Validation of dipslides as a tool for environmental sampling in a real-life hospital setting. Eur J Clin Microbiol Infect Dis.

[82] Boyce JM, Havill NL, Dumigan DG, Golebiewski M, Balogun O, Rizvani R (2009). Monitoring the effectiveness of hospital cleaning practices by use of an adenosine triphosphate bioluminescence assay. Infect Control Hosp Epidemiol.

[83] Carling PC, Perkins J, Ferguson J, Thomasser A (2014). Evaluating a new paradigm for comparing surface disinfection in clinical practice. Infect Control Hosp Epidemiol.

[84] Weber J, Henssler L, Zeman F, Pfeifer C, Alt V, Nerlich M et al. Nanosilver/DCOIT-containing surface coating effectively and constantly reduces microbial load in emergency room surfaces. *J Hosp Infect* 2023: in press. 10.1016/j.jhin.2023.01.024 36958698

[85] Mitchell BG, Dancer SJ, Anderson M, Dehn E (2015). Risk of organism acquisition from prior room occupants: a systematic review and meta-analysis. J Hosp Infect.

[86] Cohen B, Spirito CM, Liu J (2019). Concurrent detection of bacterial pathogens in Hospital roommates. Nurs Res.

[87] Bogaty C, Mataseje L, Gray A (2018). Investigation of a carbapenemase-producing *Acinetobacter baumannii* outbreak using whole genome sequencing versus a standard epidemiologic investigation. Antimicrob Resist Infect Control.

[88] Coll F, Harrison EM, Toleman MS, Reuter S, Raven KE, Blane B, Palmer B, Kappeler ARM, Brown NM, Török ME, Parkhill J, Peacock SJ (2017). Longitudinal genomic surveillance of MRSA in the UK reveals transmission patterns in hospitals and the community. Sci Transl Med.

[89] Dancer S (2019). Visualising the invisible; why cleaning is important in the control of hospital-acquired infection. Evid Based Nurs.

[90] Mora M, Mahnert A, Koskinen K, Pausan MR, Oberauner-Wappis L, Krause R, Perras AK, Gorkiewicz G, Berg G, Moissl-Eichinger C (2016). Microorganisms in confined habitats: Microbial Monitoring and Control of Intensive Care Units, operating rooms, cleanrooms and the International Space Station. Front Microbiol.

[91] Dancer SJ (2013). Floor wars: the battle for ‘clean’ surfaces. J Hosp Infect.

[92] Dancer SJ, Kramer A (2019). Four steps to clean hospitals: look, Plan, Clean and Dry. J Hosp Infect.

[93] Dancer SJ (2014). Controlling hospital-acquired infection: focus on the role of the environment and new technologies for decontamination. Clin Microbiol Rev.

[94] Dancer SJ (2011). Hospital cleaning in the 21st century. Eur J Clin Microbiol Infect Dis.

[95] Thorn RM, Lee SW, Robinson GM, Greenman J, Reynolds DM (2012). Electrochemically activated solutions: evidence for antimicrobial efficacy and applications in healthcare environments. Eur J Clin Microbiol Infect Dis.

[96] Dancer SJ, King MF (2021). Systematic review on use, cost and clinical efficacy of automated decontamination devices. Antimicrob Resist Infect Control.

[97] Graham WJ, Okomo U, Gon G, Aiken AM (2021). Cleaning neonatal units in low-resource settings: a hot-topic in Waiting?. Pediatr Infect Dis J.

[98] Ledwoch K, Dancer SJ, Otter JA, Kerr K, Roposte D, Rushton L, Weiser R, Mahenthiralingam E, Muir DD, Maillard JY (2018). Beware biofilm! Dry biofilms containing bacterial pathogens on multiple healthcare surfaces; a multi-centre study. J Hosp Infect.

[99] Dancer SJ (2022). How do biofilms affect surface cleaning in hospitals?. Hygiene.

[100] Dancer SJ (2020). How much impact do antimicrobial Surfaces really have on Healthcare-acquired infection?. Clin Infect Dis.

[101] Morawska L, Allen J, Bahnfleth W, Bluyssen PM, Boerstra A, Buonanno G (2021). A paradigm shift to combat indoor respiratory infection. Science.

[102] Hiwar W, King MF, Shuweihdi F, Fletcher LA, Dancer SJ, Noakes CJ (2021). What is the relationship between indoor air quality parameters and airborne microorganisms in hospital environments? A systematic review and meta-analysis. Indoor Air.

[103] Smith SJ, Young V, Robertson C, Dancer SJ (2012). Where do hands go? An audit of sequential hand-touch events on a hospital ward. J Hosp Infect.

[104] White NM, Barnett AG, Hall L, Mitchell BG, Farrington A, Halton K, Paterson DL, Riley TV, Gardner A, Page K, Gericke CA, Graves N (2020). Cost-effectiveness of an environmental cleaning bundle for reducing Healthcare-associated infections. Clin Infect Dis.

[105] Aiello AE, Larson EL (2002). What is the evidence for a causal link between hygiene and infections?. Lancet Infect Dis.

